# Socioeconomic resources and quality of life in alcohol use disorder patients: the mediating effects of social support and depression

**DOI:** 10.1186/s13011-020-00258-6

**Published:** 2020-02-17

**Authors:** Soo Bi Lee, Sulki Chung, Jeong Seok Seo, Won Mi Jung, Il Ho Park

**Affiliations:** 1 Department of Social Welfare, Chung-Ang University, 84 Heuksuk-Ro, Dongjak-Gu, Seoul, South Korea; 2 Department of Psychiatry, School of Medicine, Konkuk University, Chung-ju, South Korea; 3 Department of Social Welfare, Ewha Womans University, Seoul, South Korea; 4 Department of Psychiatry and Behavioral Neurosciences, Catholic Kwandong University International Saint Mary’s Hospital, Incheon, South Korea

**Keywords:** Alcohol use disorder, Depression, Quality of life, Social support, Socioeconomic resources

## Abstract

**Background:**

Quality of life (QoL) has recently attracted increased attention as a major indicator of the recovery from alcohol use disorder (AUD). This study investigated the mediating effects of social support and depression for the relationship between socioeconomic resources and QoL among people with AUD in South Korea.

**Methods:**

Patients across South Korea who had been diagnosed with AUD in the previous year (*n* = 404) and were registered at hospitals and addiction management centers were surveyed. The participants ranged in age from 19 to 65 years. Structural equation modeling was performed, using stable residence, income, stable employment, social support, depression, and QoL as predictors. Bootstrapping analysis was performed to test for mediating effects.

**Results:**

The socioeconomic resources income (*β = .297*, *p* < .001), stable employment (*β = .131*, *p* < .01), and stable residence (*β = .091*, *p* < .05) showed statistically significant and positive relationships with social support. However, none of these were significantly related to depression. Social support showed a significant and negative relationship with depression (*β = −.172*, *p* < .001). Income positively and directly influenced QoL (*β = .148*, *p* < .001). All three socioeconomic resources indirectly influenced depression through social support, which, in turn, influenced QoL. This suggests that socioeconomic resources directly influence QoL and indirectly influence it through social support.

**Conclusion:**

These findings suggest that social support has an important role in improving the QoL of people with AUD. Furthermore, socioeconomic resources, such as having a stable residence, employment, and income, are necessary for recovery from alcohol addiction.

## Background

The lifetime prevalence of alcohol use disorder (AUD) in South Korea is higher than that of many other diseases, at 12.2% for adults (18.1% for males and 6.4% for females) [1]. As of 2016, among those diagnosed with mental illness, 40.4% were diagnosed with mood disorders, 32.1% with schizophrenia spectrum disorders, and 19.3% with anxiety disorders, but only 8.1% were diagnosed with AUD [1]. There are serious, harmful effects on Korean society due to AUD, but only a small number of heavily dependent people access the limited treatment services. Public awareness of AUD in Korea is very low, and treatment efficiency and accessibility have been reduced due to trends in public perceptions regarding long-term treatment. Furthermore, the roles and functions of outpatient treatment and community mental health institutions as currently instituted are not sufficient for maintaining treatment and preventing relapse [2]. In addition, very few social rehabilitation services exist that can support recovery from AUD and successful reintegration [3].

The damage caused by AUD goes beyond harms to health, including liver disease, diabetes, high blood pressure, cardiovascular disorders [4, 5], and mental illnesses such as depression and anxiety [6]: it also impairs quality of life (QoL) in sufferers in other ways, damaging interpersonal and social roles, and this makes recovery more difficult [7]. Recovery from AUD requires a comprehensive approach that takes into account biological, psychological, and socioeconomic factors [8, 9].

Although different researchers have presented a range of conceptions of recovery from AUD, it should be considered to be a continuous, stepwise process, going beyond symptomatic improvement alone [10]. According to the New Freedom Commission on Mental Health, recovery is a healthy and efficient process that enables and empowers people with addictions to live, work, learn, and freely engage in life [11]. For AUD, improvements in QoL are arguably the ultimate goal of treatment programs and their assessment should be recognized as a major focus of research on therapeutic outcomes [12, 13]. The concept of recovery from AUD has evolved from simple observed changes in drinking patterns or decreased alcohol consumption to larger picture of overall improvements in health, relationships, and QoL [14–17]. The recovery of people with AUD in relation to QoL can be understood as a process of repair for social functions and relationships previously damaged by social and psychological challenges stemming from AUD and a return to a previous state.

It has been found that AUD sufferers who consumed less alcohol had improved physical functioning, participated in Alcoholics Anonymous (AA), had high levels of family and other social support, and had fewer emotional conflicts with their family members. Some reported relatively high levels of life satisfaction, which could be considered a proxy for QoL [12, 18]. Many South Korean studies on AUD have found that social support [19, 20], depression, and anxiety [21] are significant determinants for QoL. Depression is a common accompanying disease in people with alcohol-related disorders and is an obstacle to recovery [22, 23]. By contrast, social support, defined as any positive help [24] obtained from any of an AUD sufferer’s relationships, is a useful resource for adaptation at a preventative and therapeutic level, reduces the perception of stress, and relieves the pathological symptoms that result from it [25]. Taken together with the results of previous studies [26, 27] that indicate the association of social support with reduction in mental health problems such as depression and suicidal behavior, the importance of social support is evident. However, these studies have largely considered individual factors, such as psychological variables or drinking behaviors that affect QoL. Consequently, these studies have been limited due to their omission of antecedent factors, such as socioeconomic variables, that may affect depression in people with AUD or the social support they enjoy.

In addition to individual psychological factors, AUD is associated with important life events and problems, such as job dismissals, unstable employment, housing difficulties, poor relationships with family and others, and social deprivation [6, 7, 28]. These problems may appear either as causes or as outcomes of addiction. Recent studies on the association between drinking problems and the characteristics of vulnerable groups found that rates of alcohol abuse and dependence are higher among individuals who receive public assistance benefits, are unemployed, or have lower incomes [28].

Studies in South Korea have found that socioeconomic factors are related to problematic drinking in the general population [29–31]. Financial factors and social support also influence the state of mental health in certain socially vulnerable populations [32, 33]. However, empirical research on to the influence of socioeconomic difficulties or deprivation on the recovery process of people with AUD in South Korea has been limited.

The assessment of QoL should include the evaluation of complex relationships among aspects of physical health, psychological states, personal faith, social relationships, and the environment. For this reason, this study went beyond psychological factors and drinking behaviors in its assessment of QoL among people with AUD. The influence of socioeconomic factors and social support (indicative of social relationships), were investigated, in addition to the influence of psychological factors (e.g., depression) on QoL, using a variety of pathways. The analysis and verification of relevant path models allowed this study to develop an understanding of the recovery of patients with AUD and improvements to their QoL within a broad social context. Specifically, we examined whether socioeconomic factors were antecedents that affect the relationship between social support and depression, as well as exploring whether social support and depression mediate the relationship between socioeconomic factors and QoL in AUD.

## Methods

### Study model

**Fig. 1:**
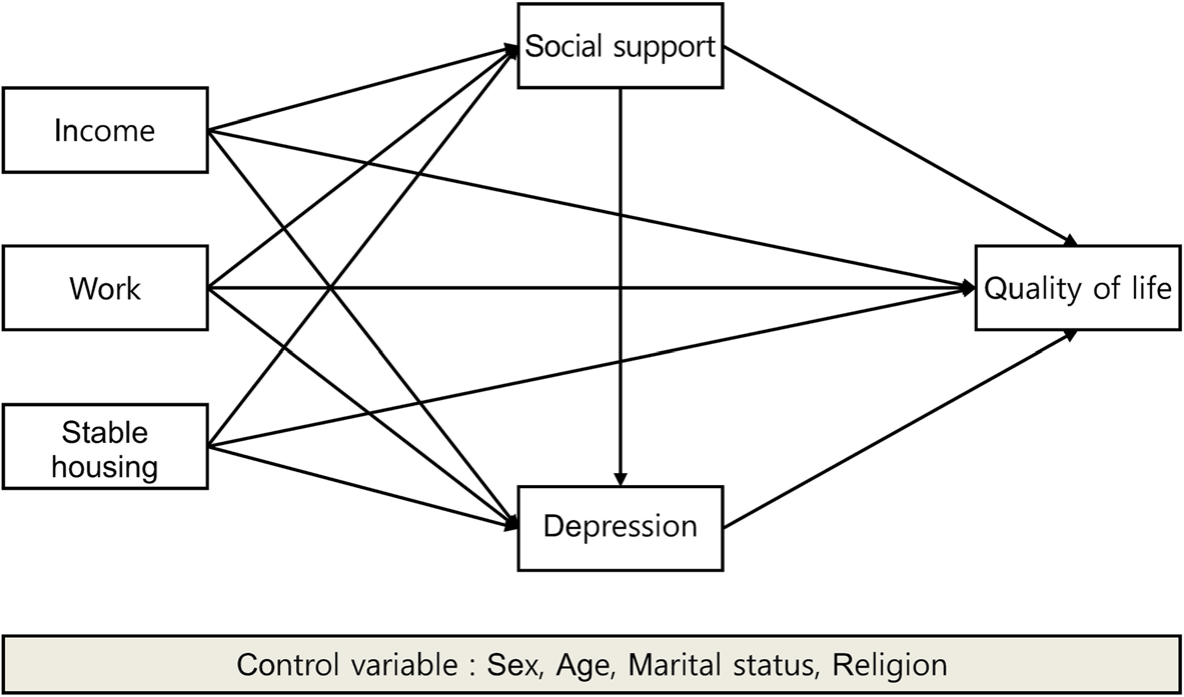
Research Model. The figure depicts the research model of the study, which examines the mediating effects of social support and depression between the socioeconomic resources and QoL among alcohol use disorder patients

The research model adopted presented how explanatory variables, such as income level, work experience, and possession of a home, affect QoL through the mediating variables of social support and depression. The study model is shown in Fig. 1.

### Sample

This study targeted men and women between the ages of 19 and 65 who were registered at 28 hospitals and 34 addiction management centers and were diagnosed as having AUD in the year before the study began. The clinicians who participated in the study employed the DSM-5 and AUDIT scales to obtain their diagnoses. A survey was conducted using one-to-one interviews and self-reporting.

The exclusion criteria were the following: being deemed unfit for the study because of severe physical illness, having a severe cognitive impairment that interfered with the perception of reality, or being otherwise assessed by clinicians as an inappropriate subject for the study. Of the 440 people who participated, 36 did not reply to more than half of the questions or were judged by the clinician to have given false responses and their data were excluded from the analysis after discussion among the researchers. Ultimately, 404 people were included in the analysis. This study was approved by the Public Institutional Review Board designated by Ministry of Health and Welfare (P01–201603–21-001).

### Main variables

#### QoL

This study used the Korean version of the World Health Organization Quality of Life Scale-Abbreviated Version (K-WHOQOL-BREF), a subjective measure of QoL over the previous two weeks with 26 questions. The self-report questionnaire includes four domains (physical health, psychological states, social relationships, and living environment), and the subscales for each domain are used to evaluate overall QoL [34]. The scores for each domain and the mean of scores for all questions within each domain were multiplied by four. The total score was the sum of the scores for each domain, and higher total scores indicated higher overall QoL.

#### Work

In this study, work describes whether a participant had held a stable job for at least 3 months over the past year, working at least 5 days per week. Response options were 0 = *no* and 1 = *yes*.

#### Housing

Housing was an indicator that a participant had a stable residence. Response options were 0 = *no* and 1 = *yes*.

#### Income

Income was an ordinal variable measuring average monthly household income. Response options were 0 = less than KRW 1,000,000, 1 = KRW 1,000,000 to less than 3,000,000, 2 = KRW 3,000,000 to 5,000,000, and 3 = more than KRW 5,000,000.

#### Social support

The Perceived Social Support Scale developed by Blumenthal et al. [35] was used to measure this variable. This scale includes 12 items: perceived social support from family (four questions), friends (four questions), and significant others (four questions), with response options given on a five-point Likert-type scale where 1 *=* strongly disagree to 5 = strongly agree. Higher total scores indicate higher perceived social support.

### Statistical analysis

The data were analyzed using SPSS 25 and AMOS 25.0, as follows. First, the participants’ demographic characteristics were assessed using descriptive statistics. Second, structural equation modeling and path analysis were performed to examine the relationships among housing, work, income, and QoL. Last, a bootstrap analysis was performed to test the mediating effects of social support and depression. Structural equation modeling, which includes statistical techniques for controlling measurement errors, facilitating the use of parameters, and enabling the statistical evaluation of the theoretical model, was considered appropriate for validation of the study model [36].

## Results

### Demographic characteristics

As shown in Table 1, the sample was 80% male, 78% unmarried, and 64.4% without religious affiliation. Slightly more participants (57.2%) had held a stable job for at least 3 months in the past year, working at least 5 days per week, than those who did not; 83.7% reported stable residence, and 52.5% reported monthly family incomes of less than KRW 1,000,000, 24.5% of KRW 1,000,000 to less than 3,000,000, 12.9% of KRW 3,000,000 to less than 5,000,000, and 10.01% of more than KRW 5,000,000. The participants’ mean age was 51.2 years.

**Table 1:** Socio-demographic characteristics: Alcohol use disorder patients (*n* = 440)

Variable		Frequency	%
Sex	Male	323	80.0
	Female	81	20.0
Marital status	Yes	89	22.0
	No	315	78.0
Religion	Yes	260	64.4
	No	144	35.6
Stable employment	Yes	173	42.8
	No	231	57.2
Housing	Yes	338	83.7
	No	66	16.3
Income	less than KRW 1,000,000	212	52.5
	KRW 1,000,000 to less than 3,000,000	99	24.5
	KRW 3,000,000 to 5,000,000	52	12.9
	more than KRW 5,000,000	41	10.1
Total		404	100.0
		Mean	Standard deviation
Age		51.16	9.65

The table represents the sociodemographic characteristics of the sample used in the study

### Correlation analysis

The correlation coefficients for the major variables used in the study ranged from .009 to .658. Because the coefficients were less than 0.7, they were considered to have met the criteria for multicollinearity diagnostic (see Additional file 1) [37].

### Path analysis

Table 2 presents the results of the path analysis model, which had an appropriate goodness-of-fit (NIB = .998, RAFI = .974, CFI = 1.000, and MESA = .000). Income (*β* = .297, *p* < .001), stable employment (*β* = .131, *p* < .01), and stable residence (*β* = .091, *p* < .05) were significantly and positively related to social support. Second, income, stable employment, and stable residence had no significant relationship to depression. However, social support did have a significant negative relationship to depression (*β* = −.172, *p* < .001). Third, income directly influenced QoL (*β* = .148, *p* < .001). The influences of stable employment and residence were not statistically significant. Path analysis found that social support (*β* = .331, *p* < .001) and depression (*β* = −.477, *p* < .001) had significant mediating effects on QoL. Patients with AUD who reported higher QoL had higher scores for social support and lower ones for depression. In brief, AUD patients’ incomes, employment, and housing were found to influence their QoL through the mediation of social support. Although socioeconomic resources did not directly influence QoL, they had statistically significant influences on depression through social support, which, in turn, influenced QoL.

**Table 2:** Path analysis results: The mediating effects of social support and depression

Predictor		Outcome	*β*	*B*	S.E.	C.R.
Income	--->	Social support	0.297***	5.700	1.009	5.652
Stable employment	--->		0.131**	5.126	1.865	2.749
Residence	--->		0.091*	4.801	2.404	1.997
Income	--->	Depression	− 0.014	−0.111	0.424	− 0.261
Stable employment	--->		0.045	0.708	0.762	0.929
Residence	--->		−0.08	−1.699	0.978	−1.737
Social support	--->		−0.172***	−0.181	0.02	−8.983
Income	--->	QoL	0.148***	1.736	0.433	4.008
Stable employment	--->		0.065	1.551	0.833	1.861
Residence	--->		0.010	0.311	1.061	0.293
Depression	--->		−0.477***	−0.722	0.053	−13.581
Social support	--->		0.331***	0.203	0.024	8.559
Sex	--->	Social support	0.071	3.441	2.146	1.603
Age	--->		0.093**	−0.249	0.094	−2.663
Marital status	--->		−0.095	4.341	2.366	1.835
Religion	--->		−0.124*	−3.833	1.769	−2.168
Sex	--->	Depression	−0.448	0.316	0.872	0.363
Age	--->		0.016***	−0.14	0.038	−3.668
Marital status	--->		−0.045	−0.856	0.962	−0.89
Religion	--->		0.102*	1.667	0.72	2.316

The table represents results of path analysis that analyzed the mediating effects of social support and depression between the socioeconomic resources and QoL among alcohol use disorder patients.

*B*, non-standardized coefficients; *β*, standardized coefficients; CR, critical ratio.

χ^2^ = 2.043, df = 4, *p* > .05 (*p* = .728).

NFI = .998, RFI = .974, CFI = 1.000, RMSEA = .000.

*** *p* < .001, ** *p* < .01, * *p* < .05

### Bootstrapping analysis

Bootstrapping was performed to investigate the mediating effects of social support and depression on the relationship between QoL and socioeconomic resources (i.e., income, stable employment, and stable residence) (Table 3). Income (.317) exerted the most significant influence on QoL, followed by stable employment (.115) and stable residence (.098). Moreover, income (.169) and stable residence (.088) had significant indirect effects on QoL. The total effect of social support on QoL was .545, and its indirect effect was significant (*p* < .01).

**Table 3:** Bootstrapping analysis for validation of effectiveness of final path analysis results

Predictor		Outcome	Total effect	Direct effect	Indirect effect	Indirect confidence interval
Income	→	QoL	.317	.148	.169	.105–.224**
Stable employment			.115	.065	.050	.000–.104
Residence			.098	.010	.088	.031–.145*
Social support			.545	.331	.214	.170–.251**

The table describes the results of bootstrapping analysis of the final path analysis, which examined the effect of predictor variables (income, stables employment, residence, and social support) on QoL among alcohol use disorder patients.

*** *p* < .001, ** *p* < .01, * *p* < .05

## Discussion

AUD causes social and employment dysfunctions, leading to a range of problems in sufferers’ families and employment [38]. Due to its high relapse rate, recovery from AUD requires continuous management throughout all aspects of life that goes beyond a focus on treatment completion alone. The concept of addiction recovery has expanded from reducing alcohol consumption to making overall improvements in health, social relationships, and QoL [14]. In particular, because AUD is a risk factor for lower QoL [16], changes in the QoL of people with AUD are relevant treatment targets. In other words, recovery from AUD could be enhanced by a focus on improving patient QoL. In turn, the hope of a better life could incentivize and motivate patient recovery [39]. Therefore, improving QoL is recognized as an important therapeutic goal [12, 13]. Psychological factors, significant relationships, and socioeconomic factors have influence on the lives of people with AUD. Socioeconomic factors, such as income, residence, and employment, might increase the risk of addiction and adversely influence people with AUD [15, 40, 41]. This study expanded the scope of previous studies and explored the ways that socioeconomic resources influence the QoL of patients with AUD through specific pathways.

This study’s findings demonstrate that having higher income, stable employment, and a stable residence increased social support for patients with AUD. Furthermore, as expected, patients who perceived higher levels of social support had lower scores for depression and higher ones for QoL. When the size of the influence was measured through bootstrapping, income level, work experience, and possession of a house were among the socioeconomic resources that most affected QoL of those with AUD; furthermore, income level and possession of a house were shown to have statistically significant indirect effects on QoL through social support and depression.

The results of the present study support previous findings showing that low social support for patients with AUD predicts lower QoL [18, 42]. Depression also predicted QoL in AUD patients. This study confirmed the findings of a previous study [15] that people with AUD and co-morbid depressive symptoms had a higher risk for relapse and a lower QoL.

Qualitative studies on the difficulties of recovery for patients from economically vulnerable groups who had AUD found that the physical and mental states that accompany addiction tend to interfere with the establishment of financial independence, which hinders recovery process. In particular, the cost of transportation to AA meetings or treatment centers could be burdensome for low-income patients. A study of QoL in people treated for addiction in Singapore found that people addicted to gambling reported a lower QoL than those who had other addictions, suggesting that financial and material hardships are significant adverse factors for QoL [39].

The study findings imply that the recovery from unstable living conditions caused by the loss of socioeconomic abilities by people with AUD will help restore QoL. Social support is a very important factor for QoL in people with AUD, either in itself or as a mediating variable. Social support can be obtained from socioeconomic resources, suggesting that it can also affect depression and improve QoL in sufferers from AUD. To facilitate recovery, addiction treatment must include intervention that takes into account the life functions of individuals whose social support has been damaged by the impact of AUD as well as the impact of a possible coexisting condition of depression. Study findings showed that socioeconomic resources did not directly influence depression, but indirectly through social support. This indicates that those suffering from AUD experienced depression not directly because they lacked socioeconomic resources, but because of the breakdown in social functions and social support resulting from lack of socioeconomic resources.

One of the limitations of this study is that the sample consists of those in treatment with a formal AUD diagnosis. Statistics show that less than 10% of those who need treatment for AUD actually receive treatment [43]. Therefore, the results should be interpreted with care when applying to people with alcohol problems in general. Second, because the study adopts a cross-sectional design, there is a predictive limitation to the relationship between socioeconomic resources and QoL. Without longitudinal data, it is difficult to establish a cause-and-effect relationship between variables.

## Conclusions

This study confirmed that socioeconomic resources directly relate to QoL, as well as indirectly through social support. In addition, socioeconomic resources were found to indirectly influence depression through social support, which, in turn, influenced QoL. Lack of socioeconomic resources could cause or be caused by addiction. Many previous studies have suggested that socioeconomically vulnerable populations are more vulnerable to addiction. Therefore, although addiction interventions must improve the level of social support for individuals and decrease depressive symptoms, ways to increase socioeconomic resources should be considered at the same time.

The results of this study demonstrate that in relation to QoL for people with AUD and their return to normal functioning and ultimate recovery, functional recovery in socioeconomic dimensions, including physical, occupational, and income factors, has a large impact on both their social support and depression, and these factors ultimately contribute to recovery, which was considered to be a return to a previous normal state that had been lost due to AUD. In short, recovery and the return to a healthy life should be considered in relation to the restoration of social functions and relationships through increasing socioeconomic resources.

## Supplementary information

**Additional file 1.** Correlations between major variables. The table describes correlation coefficients between major variables.

## Data Availability

The datasets used and analyzed during the current study are available from the corresponding author on reasonable request.

## Abbreviations

AA: Alcoholics Anonymous
AUD: Alcohol use disorder
AUDIT: Alcohol use disorders identification test
K-WHOQOL-BREF: Korean version of the World Health Organization Quality of Life Scale- Brief
QoL: Quality of life
