# Supplementary material for: Socioeconomic resources and quality of life in alcohol use disorder patients: the mediating effects of social support and depression
Source: Subst Abuse Treat Prev Policy. 2020 Feb 17;15:13. doi: 10.1186/s13011-020-00258-6 (PMC7027081; doi:10.1186/s13011-020-00258-6)
Supplement: Supplementary file 1 — Additional file 1. Correlations between major variables. The table describes correlation coefficients between major variables. [file 13011_2020_258_MOESM1_ESM.docx]

[Additional File 1](https://oup.silverchair-cdn.com/oup/backfile/Content_public/Journal/alcalc/53/5/10.1093_alcalc_agy003/1/agy003supplementaryfigure1.modelssetforpathhomogeneityanderrorcovariancehomogeneity.docx?Expires=1566015964&Signature=Q0LpFNQASEB62ezK7r6hDsdIzq7fCIoPZapV06702UGy7U4ooxRUALh5PS~JYzeJhp9VPaWsU~GX9TDyr~g~XDepBHOrdLhNqKKqKpJTU1LIUXzz8LcdycEghT~WXDQRjsTTukwqMGsOmEOhLNlnUWTbqAuWaTbpUdNXpWIuwvZaoJG3bdCJCECX0oYEMLH3hWkwqJyuuGdNgyhivQNHr58xt9smS6oyCeGT18siNapcqIr1cLkZz~zUPosm6KoI2kGlxtrWO9rZYxc0dH7Ik0XweQfOJzmvBD5hLKouFuVHDHazSuYJ41oBrSDAr~BcJOB2DZkABeHC4tm7uD6chg__&Key-Pair-Id=APKAIE5G5CRDK6RD3PGA)

**Correlations between major variables**

|  | 1 | 2 | 3 | 4 | 5 | 6 | 7 | 8 | 9 | 10 |
| --- | --- | --- | --- | --- | --- | --- | --- | --- | --- | --- |
| 1 | 1 |  |  |  |  |  |  |  |  |  |
| 2 | -.155** | 1 |  |  |  |  |  |  |  |  |
| 3 | .092 | .152** | 1 |  |  |  |  |  |  |  |
| 4 | .027 | -.065 | -.009 | 1 |  |  |  |  |  |  |
| 5 | .041 | -.093 | .264** | .066 | 1 |  |  |  |  |  |
| 6 | .138** | -.084 | .219** | .105* | .207** | 1 |  |  |  |  |
| 7 | .071 | -.199** | .421** | .091 | .388** | .253** | 1 |  |  |  |
| 8 | .135** | -.194** | .261** | -.040 | .297** | .224** | .431** | 1 |  |  |
| 9 | -.029 | -.096 | -.199** | .125* | -.099* | -.158** | -.185** | -.440** | 1 |  |
| 10 | .079 | -.064 | .291** | -.080 | .270** | .210** | .407** | .626** | -.658** | 1 |

*Note*: 1 = Gender, 2 = Age, 3 = Marital status, 4 = Religion, 5 = Stable employment, 6 = Residence, 7 = Income, 8 = Social support, 9 = Depression, 10 = Quality of life

*** = *p* < .001, ** = *p* < .01, * = *p* < .05
